# Gradually Progressive Interstitial Pneumonia Following COVID-19 in an Older Patient: A Case Report

**DOI:** 10.7759/cureus.49807

**Published:** 2023-12-01

**Authors:** Takeshi Mori, Fumiko Yamane, Chiaki Sano, Ryuichi Ohta

**Affiliations:** 1 Gastroenterology, Fuchu Hospital, Osaka, JPN; 2 Communiy Care, Unnan City Hospital, Unnan, JPN; 3 Community Medicine Management, Shimane University Faculty of Medicine, Izumo, JPN

**Keywords:** family medicine, rural hospital, post-covid sequelae, hypoxemia, prednisone, general medicine, acute respiratory distress syndrome, interstitial pneumonia, covid-19

## Abstract

Since its confirmation in Wuhan, Hubei Province, China, at the end of 2019, the novel coronavirus disease (COVID-19) has rapidly spread worldwide in multiple epidemic waves while undergoing mutations. To date, numerous individuals have been infected. Symptoms range from asymptomatic and common cold-like to acute respiratory failure and acute respiratory distress syndrome (ARDS), which can lead to death. Here, we present the case of an 81-year-old woman with a fever that persisted for more than five days after infection with severe acute respiratory syndrome coronavirus-2 (SARS-CoV-2). She underwent chest imaging that revealed complications of interstitial pneumonia presumed to be caused by COVID-19. Computed tomography (CT) findings in COVID-19 pneumonia are often nonspecific. In this case, scattered frosted shadows were observed in both lung fields, and blood tests revealed elevated Krebs von den Lungen 6 (KL-6) levels. Immediate therapeutic treatment is warranted when patients with multiple risk factors for COVID-19 present with interstitial pneumonia.

## Introduction

Coronavirus disease (COVID-19) may co-occur with various lung diseases. Moreover, the presentation of COVID-19 overlaps with that of other lung diseases, leading to increased morbidity and mortality. COVID-19, caused by the severe acute respiratory syndrome coronavirus, has rapidly spread worldwide and affected numerous individuals. While most patients present with mild disease, some develop acute respiratory distress syndrome (ARDS), leading to rapid respiratory deterioration. This emphasizes the need for an accurate and swift diagnosis of COVID-19 pneumonia [1]. Computed tomography (CT) has a high diagnostic sensitivity for COVID-19 pneumonia [1]. However, the specificity for distinguishing COVID-19 from other pneumonia types is low [2]. In cases of COVID-19 with acute exacerbations, CT can be useful to detect ARDS [3].

The presentation of COVID-19 can vary, involving the interstitium, and in rare cases, it can be progressive again after the initial resolution, accompanied by systemic symptoms such as fatigue and loss of appetite. In patients with COVID-19, typical chest CT findings include the appearance of bilateral ground glass opacity (GGO) lesions with subpleural lung predominance, exhibiting progressive consolidation of the lesions and vascular thickening in the peripheral vasculature [4]. The lesions exhibit a crazy paving appearance with reticular shading within the mottled GGO [5]. Here, we report a case of COVID-19-induced interstitial pneumonia with an initial mild presentation and progressive systemic symptoms. Through this case report, we discuss interstitial pneumonia caused by COVID-19 in the post-COVID-19 pandemic era.

## Case presentation

An 81-year-old woman was transported by ambulance to a rural community hospital after losing consciousness for approximately five minutes after going to the bathroom at home. During transportation, her level of consciousness gradually improved, and she was conscious and alert in the emergency room. No accompanying symptoms were observed. Subsequently, she was admitted to the hospital's Department of General Medicine. The patient had a history of hypertension and type 2 diabetes mellitus without any lung diseases. Her medication regimen at the time included telmisartan of 20 mg, amlodipine of 5 mg, bisoprolol of 2.5 mg, trichlormethiazide of 0.5 mg, and atorvastatin of 5 mg daily.

At presentation, the patient's vital signs were as follows: blood pressure 153/81 mmHg, pulse 73 beats/min, temperature 36.9 °C, respiratory rate 22 breaths/min, SpO_2_ 94% (O2 5 L). The volume of oxygen was gradually decreased to 1 L. A physical examination revealed a dry tongue, no respiratory murmur, and no other significant findings. The electrocardiogram showed no arrhythmias or ST changes. Additionally, echocardiography revealed no significant findings indicative of valvular disease, which suggests neuromodulatory syncope. However, she had hypoxemia with a PaO_2_ of 61.1 mmHg (O_2_, 1 L) without PCO_2_ retention (34.2 mmHg), and contrast-enhanced CT showed no evidence of pulmonary embolism or pneumonia. Laboratory tests revealed mildly elevated liver enzymes. Urea nitrogen and uric acid levels were elevated, suggesting dehydration, and CRP levels were elevated, suggesting inflammation (Table 1).

**Table 1:** Initial laboratory data of the patient CRP, C-reactive protein; eGFR, estimated glomerular filtration ratio

Marker	Level	Reference
White blood cells	5.0	3.5–9.1 × 10^3^/μL
Neutrophils	71.1	44.0–72.0%
Lymphocytes	18.8	18.0–59.0%
Hemoglobin	12.3	11.3–15.2 g/dL
Hematocrit	36.6	33.4–44.9%
Mean corpuscular volume	96.3	79.0–100.0 fl
Platelets	12.7	13.0–36.9 × 10^4^/μL
Total protein	6.6	6.5–8.3 g/dL
Albumin	3.4	3.8–5.3 g/dL
Total bilirubin	0.4	0.2–1.2 mg/dL
Aspartate aminotransferase	65	8–38 IU/L
Alanine aminotransferase	50	4–43 IU/L
Lactate dehydrogenase	255	121–245 U/L
Blood urea nitrogen	23.6	8–20 mg/dL
Creatinine	0.84	0.40–1.10 mg/dL
Serum Na	134	135–150 mEq/L
Serum K	3.5	3.5–5.3 mEq/L
Serum Cl	101	98–110 mEq/L
CRP	4.40	<0.30 mg/dL
Glucose	136	70-110 mg/dl
Creatinine kinase	375	43–165U/L
Tloponin I	0.018	0.000–0.029 ng/mL
eGFR	49.1	>60 ml/min/1.73^2^

The patient was admitted to the hospital for follow-up observation and required oxygen. The next day, the patient learned that a person who had traveled with her four days earlier tested positive for COVID-19 in the PCR test. Subsequently, the patient also tested positive for COVID-19 in the PCR test.

The patient received symptomatic treatment during the follow-up. From the second day of onset, she experienced a fever in the 37-38 °C range, which persisted until the sixth day. SpO_2_ was 88-92% on room air. Due to persistently decreasing oxygenation and a decline in appetite, further examination was conducted to identify her symptoms. Physical examination revealed inspiratory crackles in both lung fields, but no costovertebral angle tenderness or arthritis was observed.

**Figure 1:**
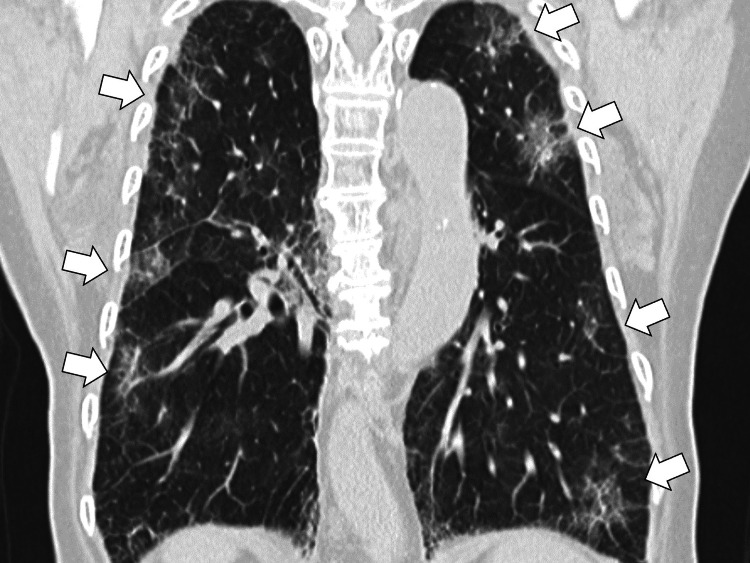
A chest computed tomography revealing multiple infiltrative shadows in the bilateral lungs (white arrows)

Blood tests revealed an elevated inflammatory response, with a 10.77 mg/dL C-reactive protein (CRP). Urinalysis results were negative for bacteria, and a urinary tract infection was ruled out. A chest CT revealed multiple infiltrative shadows in both lungs (Figure 1).

Because Krebs von den Lungen 6 (KL-6) levels were elevated by 573 U/ml (Reference, 105.3-401.2), and the tests for anti-nuclear antibodies and anti-neutrophil cytoplasmic antibodies were negative, COVID-19-induced interstitial pneumonia was diagnosed. Therefore, on day 7 of admission, prednisone (30 mg/day) was initiated for COVID-19-induced interstitial pneumonia. After starting prednisone, appetite improved, and fever resolved on day 9 of hospitalization. On the 12th day of hospitalization, blood tests showed an improved inflammatory response with a CRP of 1.14. The patient was discharged after rehabilitation. In the follow-up, her KL-6 decreased within the normal range, and the prednisolone was tapered gradually to 5 mg within two months.

## Discussion

This case report highlights general medicine's critical role in managing COVID-19 and the associated interstitial pneumonia. The initial discovery of COVID-19 in patients with hypoxemia underscores the importance of vigilance and comprehensive assessment in general medical practice [6]. During hospitalization, the patient's persistent fever, hypoxemia, and decreased appetite necessitated thorough investigations, including chest CT and blood tests. These investigations, integral to general medical practice, led to the diagnosis of COVID-19-induced interstitial pneumonia, characterized by scattered frosted shadows in the lung fields and elevated KL-6 levels [7].

A holistic approach to general medicine was pivotal in managing this case, as it enabled the integration of symptom management, diagnostic imaging, and laboratory findings [8,9]. This integrative approach is crucial in COVID-19, where presentations range from mild common cold-like symptoms to severe multi-organ failure, often accompanied by a cytokine storm [10]. General medicine provides a framework for managing diverse presentations, emphasizing early detection and comprehensive care.

Treatment with prednisone and the significant response observed highlight the role of general medicine in implementing and monitoring pharmacological interventions. Steroids, immunosuppressive drugs, and antiviral therapy effectively manage moderate-to-severe COVID-19 pneumonia [11,12]. This case illustrates the need for general practitioners to be adept at prescribing and managing these therapies, especially considering the variability in treatment protocols across different regions.

Management of post-COVID-19 sequelae, such as interstitial lung disease, has been observed in some patients, further emphasizing the role of general medicine. Post-discharge monitoring and treatment adjustments are crucial, as evidenced by the improvement in patients receiving steroid therapy for interstitial lung disease [11,13]. General medical physicians play a vital role in these patients' long-term follow-up and management, ensuring continuity of care and optimizing recovery through continual dialogue in communities [14]. In severe cases of COVID-19-induced interstitial pneumonia, where patients do not respond adequately to antivirals and steroids, general medicine physicians must consider the addition of immunosuppressive agents, such as tocilizumab [15,16]. General physicians should collaborate with multiple medical professionals and update evolving treatment modalities in general medicine.

## Conclusions

This case demonstrates the acute progression of COVID-19-associated interstitial pneumonia in an older patient treated effectively with prednisolone. COVID-19 can cause various complications in acute and chronic phases, which should be detected promptly and approached by general physicians as front-liners of healthcare. When COVID-19 patients' respiratory conditions may progress after initial recovery, the development of interstitial pneumonia should be considered in differential diagnosis and treated quickly for better recovery.
